# Engineering an Integrated Bioprocess to Produce Human Dental Pulp Stem Cell-Alginate-Based Bone Organoids

**DOI:** 10.3390/ijms26094348

**Published:** 2025-05-03

**Authors:** Mauricio Zamorano, Cristobal Aguilar-Gallardo, Aloyma Lugo, Luis Jimenez, Jorge G. Farias, Athanasios Mantalaris

**Affiliations:** 1 Chemical Engineering Department, Universidad de La Frontera, Temuco 4811230, Chile; aloymalugo@gmail.com (A.L.); jimenezalmaguer93@gmail.com (L.J.); jorge.farias@ufrontera.cl (J.G.F.); 2 Biological Systems Engineering Laboratory, Department of Chemical Engineering, Imperial College London, London SW7 2AZ, UK; cristobal_aguilar@iislafe.es; 3 Instituto de Investigación Sanitaria Hospital La Fe, Valencia 46026, Spain; 4 Bioprocess Systems Engineering Group, Trinity College Dublin, Dublin A94 X099, Ireland

**Keywords:** bone tissue engineering, alginate organoids, perfusion, bioreactor, human dental pulp stem cells

## Abstract

Bone tissue engineering (BTE) emerged as a practical approach to tackle prosthetic industry limitations. We merge aspects from developmental biology, engineering and medicine with the aim to produce fully functional bone tissue. Mesenchymal stem cells have the capability of self-renewal and specific lineage differentiation. Herein lies their potential for BTE. Among MSCs, human dental pulp stem cells have a higher proliferation rate, shorter doubling times, lower cellular senescence, and enhanced osteogenesis than hBM-SCs under specific conditions. In addition, these cells are readily accessible and can be extracted through a subtle extraction procedure. Thus, they garner fewer moral concerns than most MSCs available and embody a promising cell source for BTE therapies able to replace hBM-MSCs. Interestingly, their study has been limited. Conversely, there is a need for their further study to harness their true value in BTE, with special emphasis in the design of bioprocesses able to produce viable, homogenous bone constructs in a clinical scale. Here, we study the osteogenic differentiation of hDPSCs encapsulated in alginate hydrogels under suspended culture in a novel perfusion bioreactor. The system is compared with traditional 3D static and fed-batch culture methodologies. The novel system performed better, producing higher alkaline phosphatase activity, and more homogeneous, dense and functional bone constructs. Additionally, cell constructs produced by the in-house-designed system were richer in mature osteoblast-like and mineralizing osteocyte-like cells. In conclusion, this study reports the development of a novel bioprocess able to produce hDPSC-based bone-like constructs, providing new insights into hDPSCs’ therapeutic potential and a system able to be transferred from the laboratory bench into medical facilities.

## 1. Introduction

Bone tissue engineering (BTE) emerged as a practical approach to tackle prosthetic industry limitations. By merging aspects from developmental biology, engineering and medicine, it is possible to produce fully functional bone equivalents for research and therapeutic purposes. Mesenchymal stem cells (MSCs) have the capability of self-renewal and/or specific lineage differentiation [1]. Herein lies their potential for BTE. It has been found that biochemical stimuli from a multitude of drugs, cytokines, hormones and growth factors, e.g., bone morphogenetic proteins (BMPs), fibroblast growth factors (FGFs), and parathyroid hormone (PTH) [2,3,4,5], as well as biophysical stimuli from different sources, e.g., mechanical stimuli, shear stress, infrared light (IR), and magnetism [6,7,8,9], trigger MSCs commitment into osteogenic lineage. Among MSCs, human bone marrow-derived stem cells (hBM-SCs) have always been the gold-standard cell source for BTE solutions. Yet, since their discovery over 20 years ago, human dental pulp stem cells (hDPSCs) have been identified as a promising cellular adjunct to traditional hBM-SCs, especially for craniofacial and dental tissue regeneration. The predilection of DPSCs for these applications stems from their neural crest origin, which confers a distinct propensity for osteogenic and chondrogenic differentiation. Clinical trials and in vitro studies have underscored this potential, revealing that DPSCs, when cultured in appropriate osteoinductive media, not only match but occasionally surpass the osteogenic capabilities of BMSCs. A study by Lee et al. [10] demonstrated their comparable, if not superior, bone regeneration capacities in a rabbit calvarial bone defect model, thus highlighting the utility of DPSCs in localized bone growth scenarios. Their potential for BTE has been further confirmed by several authors [11,12,13]. Adding specific scaffold technologies and growth factors also plays a critical role in maximizing the regenerative potential of DPSCs [14]. These materials support the specific matrix environment, enhancing the functionality of DPSCs in tissue engineering.

hDPSCs are mesenchymal precursor cell marker (STRO-1)/receptor tyrosine kinase (c-Kit)-positive ectomesenchymal stem cells that share most features from MSCs, including multilineage differentiation, immunosuppressive and regenerative functions. Also, these properties have been seen to be maintained regardless of the age of the donor they are sourced from [15]. In addition, they are readily accessible and can be extracted through minimally invasive procedures from disposable byproducts of routine dental interventions [16,17]. Thus, they embody a promising cell source for BTE therapies able to replace hBM-MSCs. Interestingly, their study has been limited, mostly focusing on their potential for dentistry. Conversely, there is a need for their further study to harness their true value in BTE, with special emphasis in designing bioprocesses to produce viable, homogenous bone organoids in a clinical setting. In addition, there is a need to provide proper ex vivo models that enable closing the gap between these, and preclinical studies, thus minimizing the reliance on ethically challenging and expensive animal models and clinical settings when testing new therapies and/or therapeutics.

## 2. Results

Viability and osteogenic differentiation were assessed by measuring proliferation, gene expression, ALPase activity, SEM microscopy, histology, immunostaining and mineralization through different techniques.

### 2.1. The Cell Viability of the hDPSC 3D Osteogenic Bioprocess

**Figure 1:**
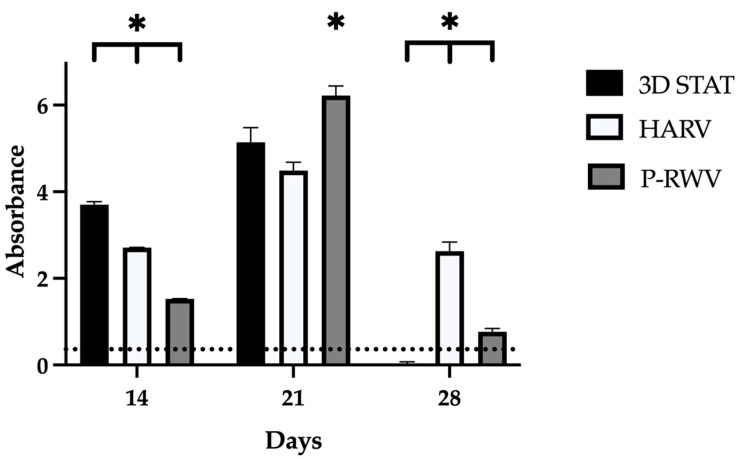
Measurement of hDPSC proliferation by CCK-8 cell viability assay scored by HARV, P-RWV and 3D STAT through 28 days of culture. For each group, *n* = 3. * indicates statistical significance comparing bioprocesses for each time point. The dotted line represents the values at the beginning of culture.

**Figure 2:**
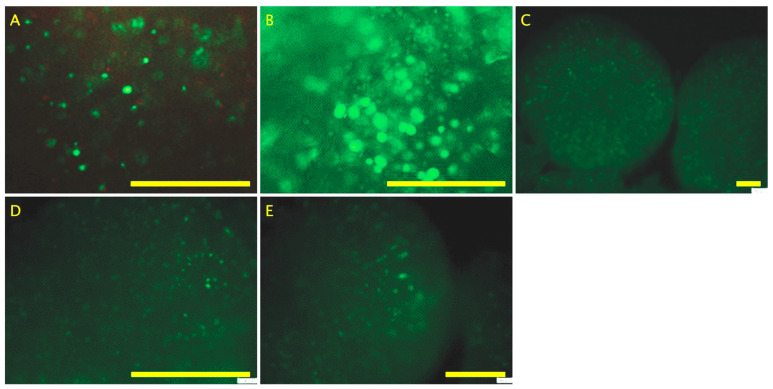
Live/dead assay (**A**) at day 0, (**B**) day 7, (**C**) day 28 for cells seeded in P-RWV, (**D**) day 28 for cells seeded in HARV, and (**E**) day 28 for cells seeded in 3D STAT. Scale bar = 200 μm.

Growth kinetics of hDPSCs showed that by day 21, P-RWV yielded significantly higher cell numbers than the other bioprocesses, with an enhancement of approximately 7 fold, compared with 5.8 and 5 fold achieved by HARV and 3D STAT, respectively. Although by day 28 MTS activity was decreased, it is believed that this is the natural course for bone tissue, when osteoblasts transition to osteocytes. Osteocytes represent approximately 90% of bone cells, and although these develop from osteoblasts, only a fraction of osteoblasts (approximately 15%) will transition to osteocyte-like cells. In addition, excessive mineralization of bone tissue, may be accompanied by apoptosis. Thus, this reduction in MTS activity may be related to interference in the measurement by this transition and/or the regulation of mineralization. This may explain why 3D STAT provided the higher decrease in MTS, where lacunae have become vacant and cells have relocated to the scaffold surface. Osteocyte transition would regulate cell density, and define spatial patterning of osteocytic lacunae, osteocyte networks and osteoblasts in the constructs. Alternatively, the MTS assay has been associated with some limitations that impair its accuracy (e.g., incubation time required may vary considering changes in the matrix and optical and/or chemical interference). Hence, it should be supported by complementary assays. Thus, mineralization or enzymatic silencing related to differentiation could direct the MTS reaction. Live/dead assay provided such complementary information. This assay utilizes a combination of calcein AM (green fluorescence) and propidium iodide (red fluorescence) to differentially stain live and dead cells, respectively. At the conclusion of the culture period, the predominance of green fluorescence observed under the fluorescence microscope confirmed that a substantial majority of the cells remained alive and retained normal physiological function (Figure 1 and Figure 2).

### 2.2. Hematoxylin and Eosin Staining

**Figure 3:**
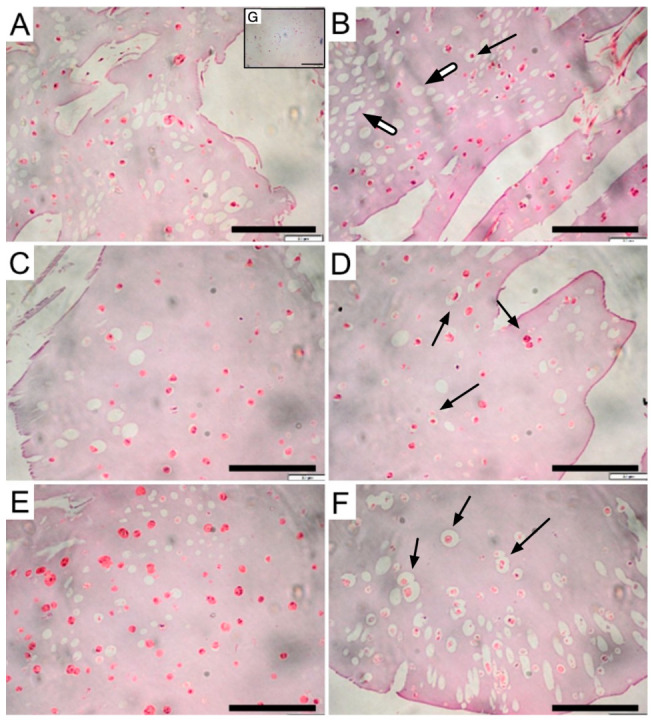
H&E images showing the morphology of alginate beads: pictures displaying staining of beads produced by 3D STAT at (**A**) day 14 and (**B**) day 28 of culture; by HARV at (**C**) day 14 and (**D**) day 28 of culture; by P-RWV at (**E**) day 14, (**F**) day 28, and (**G**) day 0 of culture. Scale bar = 200 μm. Arrows display examples of lacunae and white shaft arrows empty lacunae.

Hydrogel organoids were examined by H&E. As shown in Figure 3, samples displayed heterogeneous morphology and behaved differently. However, the three groups showed gradual bone formation. At day 0, most cells appeared undifferentiated and homogenously scattered through the beads. As time passed, the size and shape of cells, together with their surroundings changed; somewhat round lacunae started showing around single cells, which is a signal of cell differentiation. These lacunae, when cells matured, became more elongated (osteocyte lacunae). The 3D STAT showed fair formation of round-shaped lacunae by day 21 and increased by day 28 (Figure 3). Interestingly, empty lacunae started appearing (day 14) and cells were found attached to the flask surface (picture in Supplementary Materials). HARV showed less differentiation than STAT. Additionally, fewer cells were visible in the hydrogels. P-RWV displayed the production of a more mature morphology. By day 21, cells fully populated scaffolds and round lacunae was formed in all the beads except from the most inner area of the beads. By day 28, elongated lacunae were seen in a large fraction of beads.

### 2.3. Surface Analysis

**Figure 4:**
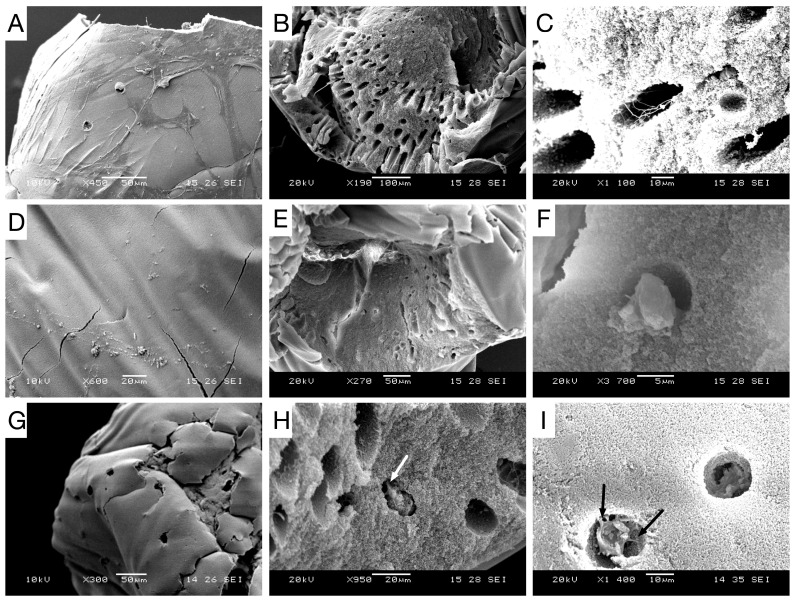
SEM pictures of cultured scaffolds in the differentiation medium after 28 days showing characteristic bone features: Pictures (**A**) displays cell constructs produced by 3D STAT after 14 days and (**B**,**C**) 28 days. Pictures (**D**) displays cell constructs produced by HARV after 14 days and (**E**,**F**) 28 days. Pictures (**G**) display cell constructs produced by P-RWV after 14 days and (**H**,**I**) 28 days. Arrows display canaliculi-like features.

SEM pictures showed the presence of non-stoichiometric mineralized deposits within organoids after osteogenic culture. Nodules were mainly composed of calcium and phosphorous (proved by X-ray microanalysis). Additionally, lacunae and morphogenesis of the osteoblast-like cells were clearly observed in these pictures. By day 28, lacunae are distributed in large portions of scaffolds. In P-RWV culture, osteocyte-like cells can be morphologically recognized by their canaliculi (Figure 4E,F). In STAT, a large proportion of lacunae were empty (in accordance with H&E) (Figure 3). HARV showed cell heterogeneity and less mature neo-tissue (Figure 4C,D).

### 2.4. Mineralization Analysis

We addressed whether hDPSCs were capable of forming mineralized bone nodules after bioprocessing. To confirm the produced bone tissue, histology and immunostaining were performed (ARS and VK). Additionally, quantitative comparisons of mineralization were performed by ALPase activity, chemical analysis with ATR-FTIR spectroscopy, X-ray microanalysis and qPCR.

### 2.5. Histological Analysis and Immunostaining of Mineralization

**Figure 5:**
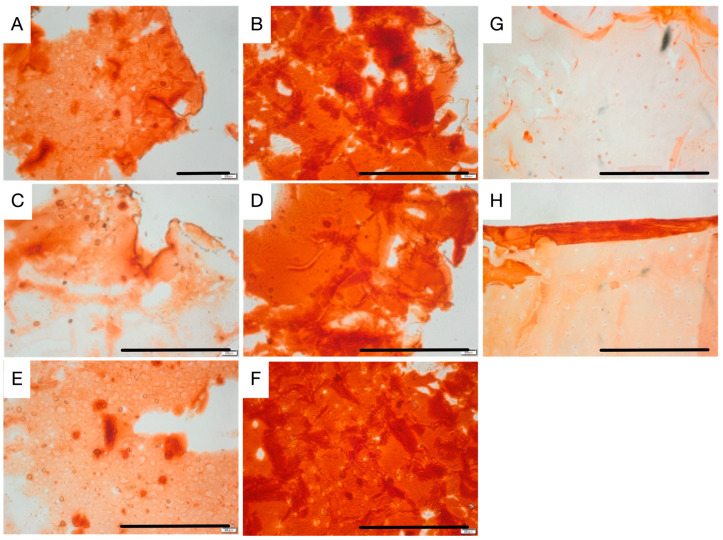
Alizarin red staining of beads produced: 3D STAT at (**A**) day 14 and (**B**) day 28 of culture; HARV at (**C**) day 14 and (**D**) day 28 of culture; P-RWV at (**E**) day 14 and (**F**) day 28 of culture; (**G**) day 0 of culture and (**H**) negative control. Scale bar = 200 µm.

**Figure 6:**
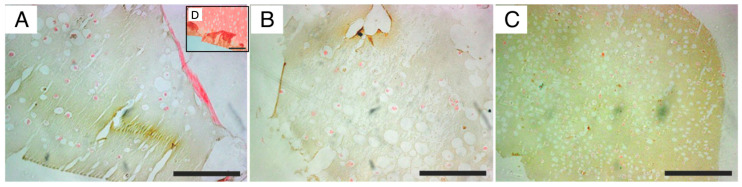
Von Kossa staining of beads after 28 days bioprocessed in: (**A**) 3D STAT, (**B**) HARV, and (**C**) P-RWV; (**D**) negative control. Scale bar = 200 μm.

The resulting organoids were examined by ARS and VK staining. As shown in Figure 5 and Figure 6, calcified deposition (ARS and VK) was widely spread in beads, with increased nodule numbers over time. STAT and P-RWV produced larger nodules through the 28 days, while HARV produced smaller nodules in higher quantities. Although VK results do not show mass deposition (black color), bead constructs, especially P-RWV, formed plenty of dispersed depositions of calcium (grey color) (see Figure 6).

**Figure 7:**
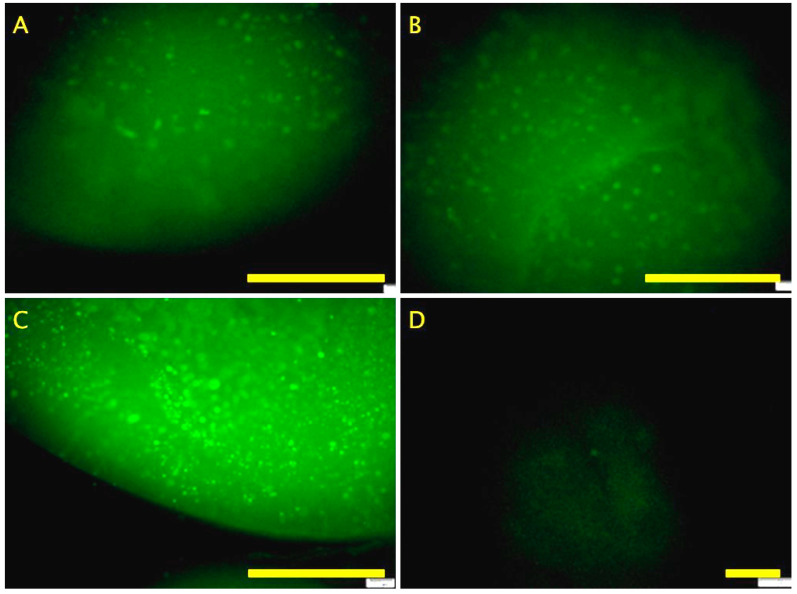
Fluorochrome labelling of bone constructs for osteocalcin of beads differentiated after 28 days in culture of (**A**) HARV (**B**) 3D STAT, (**C**) P-RWV and (**D**) Day 0. Scale bar = 200 μm.

For further confirmation of osteogenesis, hydrogel organoids were stained with anti-osteocalcin antibody (Figure 7). By day 28, all samples were positive for osteocalcin. P-RWV showed higher intensity.

### 2.6. Quantitative Comparison of Mineralization

**Figure 8:**
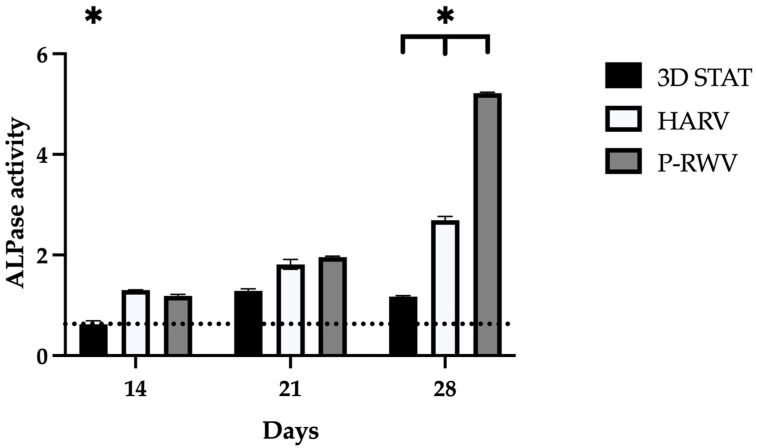
Measurement of alkaline phosphatase activity by colorimetric assay, scored by HARV, P-RWV and 3D STAT through 28 days of culture. For each group, *n* = 3. * indicates statistical significance between the three groups for each timepoint. The dotted line represents the values at the beginning of culture.

ALPase activity increased with time, with STAT showing the lowest increase. Additionally, P-RWV achieved significantly higher activity than the other bioprocesses, 7.3 fold its initial activity (Figure 8).

**Figure 9:**
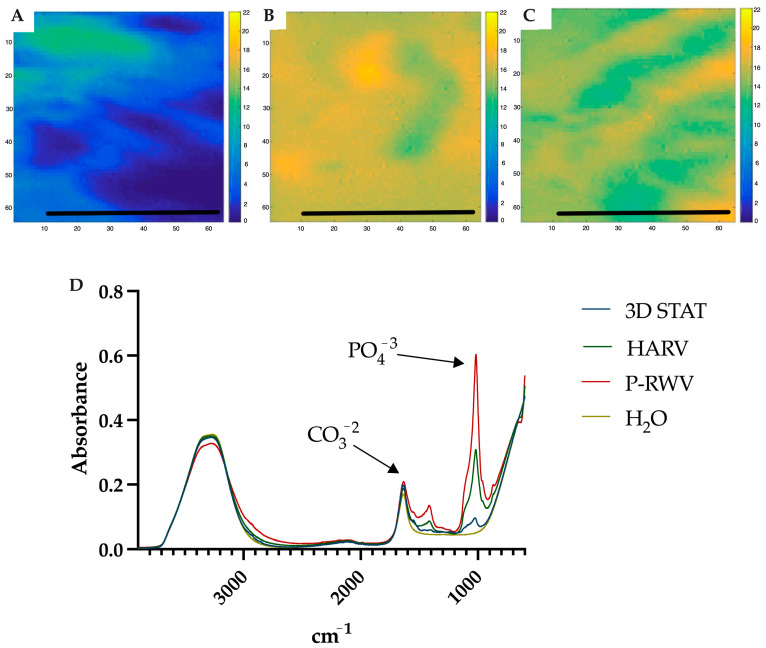
ATR-FTIR imaging of (**A**) 3D STAT, (**B**) HARV and (**C**) P-RWV after 28 days of culture. These images where obtained with a field of view of 650 × 580 μm^2^. Factor analysis was applied to the region of 1110–990 cm^−1^. Scale bar = 200 μm. (**D**) ATR-FTIR spectra comparison for the three bioprocesses after 28 days of culture.

**Figure 10:**
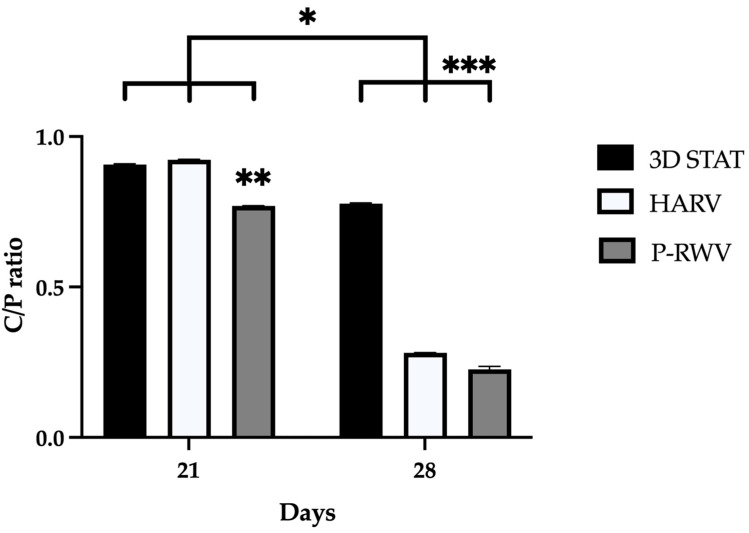
Measurement of the relationship between the organic and inorganic fractions of bone organoids by ATR-FTIR. The C/P ratio of organoids for different sampling days. For each group, *n* = 3. * indicates statistical significance between the three groups for each time point. ** and *** denote significant difference between samples in the same timepoints.

**Figure 11:**
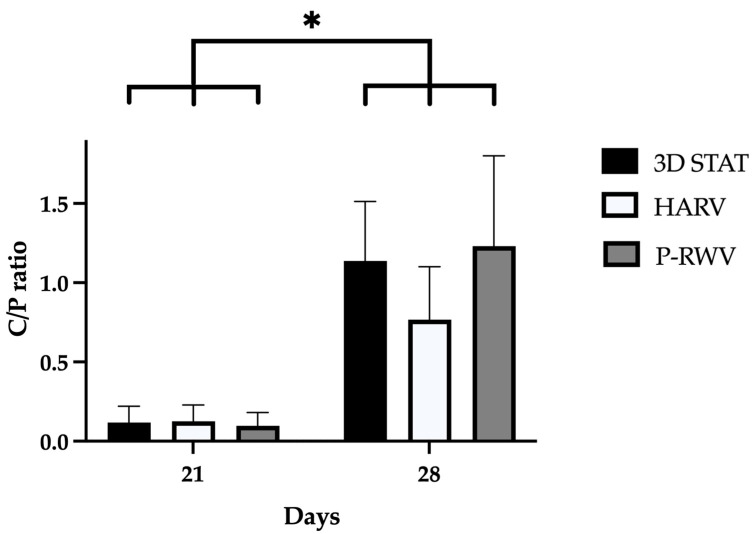
Measurement of hydroxyl apatite quality by X-ray microanalysis. Standardized Ca/P ratios characteristic of bone scored by HARV, P-RWV and 3D STAT through 28 days of culture (*n* = 3). * indicates statistical significance between the three groups between timepoints. No significant differences were seen in the three groups for each timepoint.

Spectra from ATR-FTIR and X-ray microanalysis confirmed that the comparison of mineralization was based on true calcified HA components. They were characterized in ATR-FTIR by measuring the ratio between the specific spectral bands at 1415 cm^−1^ (carbonates) and 1035 cm^−1^ (phosphates). This carbonate to phosphate (C/P) ratio is characteristic of the pure HA and collagen (COL) fractions of human bone, respectively (Figure 9 and Figure 10). In X-ray microanalysis, samples were characterized by the calcium to phosphate ratio (Ca/P). P-RWV achieved better results in both analyses, with a stronger signal in the mentioned bands and showing an average Ca/P ratio closer to the values of regular human bone (Figure 11).

### 2.7. Quantitative Polymerase Chain Reaction (qPCR)

**Figure 12:**
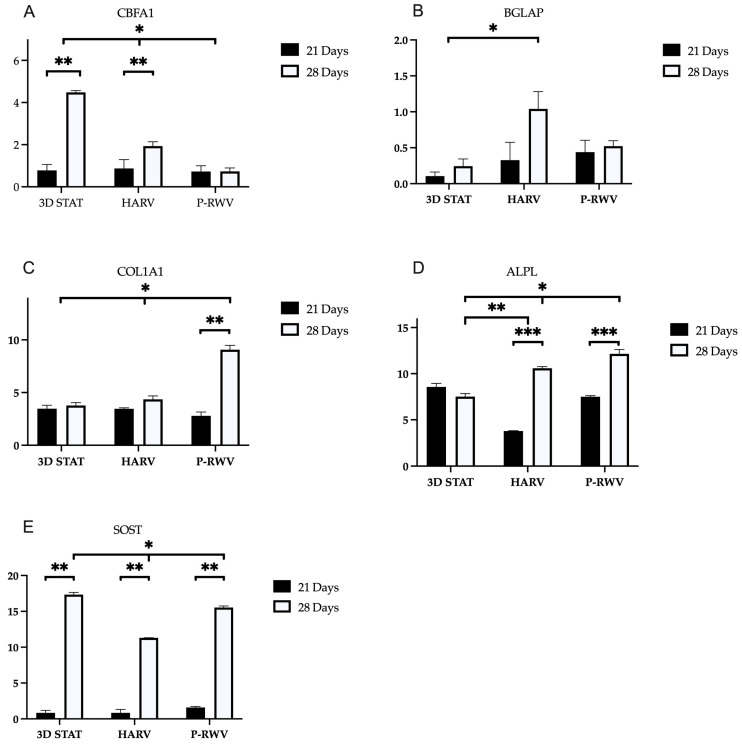
Relative gene expression for the osteogenic markers (**A**) RUNX2, (**B**) BGLAP, (**C**) COL1A1, (**D**) ALPL and (**E**) SOST, for the three bioprocesses for 28 days of culture under differentiation bioprocessing (*n* = 3). * indicates statistical significance comparing day 21 and day 28 of culture. ** and *** denote significant difference between samples in the same timepoints.

Differentiation was also characterized by distinct cellular phenotypes. The expression of genes Runx2/CBFA1, osteocalcin (BGLAP), alkaline phosphatase (ALPL), collagen type I (COL1A1) and sclerostin (SOST) can be seen in Figure 12.

Expression profiles were indicative of successful osteogenic differentiation, especially when going from day 21 of induction to day 28. P-RWV had a steady expression of Runx2 and upregulation of COL1A1, BGLAP, ALPL and SOST in the last 7 days of culture. HARV had a similar profile with softer upregulation. STAT had a steady expression of ALPL and COL1A1 and upregulation of Runx2, BGLAP and SOST in the last 7 days of culture.

The metabolic activity of hDPSCs cultured in the perfusion-RWV bioreactor, HARV bioreactor or 3D static culture under osteogenic differentiation was evaluated by measuring during the 28-day culture, the time-course concentration of key substrates and metabolites in the culture medium. This information is included as Supplementary Material.

## 3. Discussion

With the aim to produce bone-like constructs, several studies in 2D and 3D platforms have been performed, showing the relevance of the combination of MSCs, scaffolds, cues and bioreactors [18,19,20,21,22,23,24,25], displaying success in osteogenic differentiation. Nonetheless, these efforts have been insufficient and these bioprocesses often produce cellular constructs with heterogeneous populations of cells. The in vivo microenvironment is a complex and dynamic setting, in which transport of nutrients and physical stimuli happens naturally and optimally. In 2D/3D STAT, this does not happen and limited nutrient and oxygen transport from the culture medium to the innermost layer of the scaffolds, along with lack of proper physical cues represent the standard. To overcome these strains, medium perfusion has been introduced into the design of culture vessels. Hence, providing dynamic exchange of nutrients and oxygen and a source of controlled shear [26].

Herein, we developed an integrated bioprocess based on hDPSC organoids and dynamic culture in an in-house-designed perfusion bioreactor, resulting in the production of functional mineralized constructs resembling the histo-architecture of bone (bioreactors can be found in the Supplementary Material provided).

Our design produces a gentle suspension culture by providing laminar flow of the medium and rotation of the vessel, thus minimizing hDPSCs exposure to shear and turbulence, while improving diffusion of nutrients, and removing undesirable cellular byproducts. In this setting, we explored the complex dynamics of cellular differentiation, which is influenced by several critical factors. Cell source is paramount as different tissues can inherently possess distinct propensities for differentiation [27]. For example, SCs from different sources can behave differently under identical conditions. Donor age also affects outcomes, as older cells tend to grow slower and differentiate less efficiently due to senescence and DNA damage. Furthermore, medium composition can dramatically influence cellular outcomes [28]. Our experiments utilized a standardized medium optimized for the osteogenic differentiation of hDPSCs, ensuring that the medium’s concentration was conducive to replicating physiological conditions as closely as possible. In addition, our experimental design included multiple replicates and was structured to minimize variability. We conducted three independent experiments, each with three replicates, to ensure that our findings were consistent and could be reliably reproduced. The cells used were pooled from multiple donors to mitigate the impact of individual genetic variability, and all experiments utilized cells at the same passage number to reduce variations due to cellular aging or passage effects. This rigorous approach asserted that the observed results are robust and reliable, providing valuable insights into the differentiation behaviors under dynamic culture conditions influenced by the aforementioned factors.

We have explored the significant role of shear stress within a dynamic culture environment, facilitated by an in-house-designed perfusion bioreactor. This bioreactor meticulously controlled the flow of the culture medium across hDPSCs embedded within organoids. A rotating culture vessel provided an environment wherein organoids were maintained in perpetual fall, thus providing a simulated microgravity that improved diffusion of nutrients, while inducing laminar flow of media [29]. Shear stress, induced by this laminar flow, is a pivotal mechanical force that modulates key cellular behaviors critical to TE. Our findings reveal that the application of shear stress enhanced the osteogenic differentiation of hDPSCs. This is evidenced by the upregulation of osteogenic markers such as ALPase and osteocalcin, alongside the increased deposition of mineralized matrix components. These suggest that shear stress not only supports but also accelerates the maturation of hDPSCs into osteoblast-like cells. The mechanism behind these effects involves the activation of mechanotransduction pathways within cells, which are known to govern proliferation, differentiation, and mineralization of SCs [2]. By mechanically stimulating these pathways, our dynamic culture environment mimics physiological conditions that are otherwise challenging to replicate in static culture systems [19].

The in-house-designed P-RWV demonstrated higher cell proliferation and viability than the compared bioprocesses. The differences found, as mentioned, may have their roots in mass transfer, as well as in the delicate mechanical stimulation caused by the hydrostatic pressure of the suspended culture in the microgravitational environment provided by the rotation of the vessel [30]. In the case of STAT, the comparatively lower cell viability might be produced by nutrient diffusional limitations. Additionally, if rate of nutrient consumption from the organoid surface is faster than their replenishment inside, then a concentration gradient is generated and the seeded cells may experience malnutrition or decreased concentrations of growth factors vital for their long-term viability/differentiation. This is similar to what has been seen by other authors when static culture is coupled with ¨thick¨ scaffolds (more than 300 µm) [31]. If the only transport mechanism available is molecular diffusion, then cellular metabolic requirements cannot be satisfied, while perfusion enhanced nutrient transport, thereby decreasing the existing concentration gradients [21,32,33]. In the case of HARV, no significant differences with STAT were found, and rotation alone did not produce a physiologically relevant transport improvement, hence the mechanical stimuli from perfusion could be key.

Cell growth and expression in 2D STAT has three distinct phases: (1) high proliferation and development of collagenous ECM (10 to 12 days); (2) matrix maturation, downregulation of proliferation and upregulation of ALPase activity (12th to 18th day); (3) high mineralization and further decrease in proliferation, declination of ALPase and induction of osteocalcin expression (16th to 20th day) [34]. Similar behavior can be seen in the three platforms assessed, with a time delay, and this could be associated with the 3D environment. The decreased cellularity observed at the end of experiments on organoids cultured in the three bioprocesses is in accordance with the apoptotic behavior observed of mature osteoblasts residing in mineralized nodules [21]. Although the live/dead assay showed that by day 28, organoids were fully populated of living cells, which suggests that the decrease in MTS assay scores could be result of the mentioned reduction in proliferation and enzymatic activity, the product of the transition from osteoblast-like to osteocyte-like cells.

The bone neo-tissue formed within organoids consisting of viable and metabolically active cells, which were evaluated by measuring pH, glucose, glutamine, lactate, ammonia and oxygen kinetics in the osteogenic medium during cultures. It has been reported that the profile of pH and these metabolites can be used to modulate cell metabolism [35,36]. Glucose and glutamine are key energy sources for animal cells. In this study, glucose consumption kinetics were comparable between bioreactors, showing a gradual decrease indicative of sustained cell growth. However, in 3D STAT, glucose was rapidly depleted, likely due to a low initial concentration. A high-glucose medium could enhance the process, though it may increase lactate production, which must be monitored to avoid cytotoxicity (>16 mmol/L) [37]. Lactate remained undetectable in both systems, suggesting oxidative metabolism and possible cell differentiation, except in 3D STAT, where it peaked at 12 mmol/L upon glucose exhaustion. Glutamine and ammonia showed similar trends, decreasing initially then rising, with ammonia levels remaining non-toxic. pH declined over time, correlating with metabolite accumulation, but stayed within physiological limits (7.0–7.4) [38]. Oxygen remained at normoxic levels across all processes, supporting differentiation.

For validation of osteogenic differentiation, organoids were characterized throughout the differentiation process by histology, immuno-staining and SEM microscopy. Histological H&E staining of sectioned organoids at day 0 showed that most cells appeared scattered without having formed an extracellular matrix. After, they displayed well-distributed tissue growth for both bioreactors and heterogeneity for STAT. Morphogenesis of the osteogenic cells until achieving maturation was seen in both the perfusion and STAT by the emergence of osteocyte lacunae-like features. This is not so clear in the HARV culture. However, the presence of vacant lacunae in STAT revealed that cells may have left scaffolds. Proof of this is seen in one of the supplementary images, where approximately confluent cells growing attached to the flask are seen. A probable reason may be poor diffusive transport nutrients to the inner area of organoids, forcing cells to migrate to the exterior of organoids, and further to the flask surface [21]. Further, this could be behind the low MTS and ALPase activity achieved at the end of STAT. ARS staining displayed a progressive differentiation and a gradual enhancement in mineral deposition by the three bioprocesses. The ARS quantitative analysis was consistent with qualitative results and both bioreactors showed better performance than STAT, and VK confirmed the mineralization by showing disperse deposits of calcium through the organoids for the three bioprocesses. Positive immunostaining for osteocalcin for the three bioprocesses is a hallmark of their differentiation state. Nonetheless, there was clearly a higher expression in P-RWV. The surface of organoids was analyzed with SEM microscopy; here, the morphogenesis of the hDPSCs could be perceived at different levels for the three bioprocesses—P-RWV showed higher neo-tissue maturation, seen by the many HA crystals found, the osteocyte lacunae and the presence of osteocyte-like cells with canaliculi-like features. STAT showed more maturation than HARV. However, most of the quantitative analysis suggests that this maturation is only in the surface of the construct, whereas HARV produced more homogeneous constructs.

ATR-FTIR of normal human bone shows carbonate bands at wavenumbers 870, 1415 and 1470 cm^−1^, and phosphate bands at wavenumber 565, 605 and 1035 cm^−1^. Carbonate bands are distinctive of the bone organic component (mainly collagen) and phosphate bands of the inorganic fraction (HA), and their C/P ratio is a characteristic biogenic signature for human bone. However, it is recommended to use the phosphate peak of 1035 and the carbonate peak of 1415 to calculate the C/P ratio. When these wavenumbers are used, the human bone C/P ratio is 0.23 [39]. When differentiation starts, the C/P ratio is high, and it decreases with differentiation and HA formation. STAT produced a high C/P value, showing poor differentiation. Both bioreactors provided adequate results, with HARV achieving ≈ 0.28 and P-RWV achieving ≈ 0.23, the exact value described for human bone in the literature. ATR-FTIR imaging confirmed these results.

X-ray microanalysis (SEM/EDS) of HA shows the elemental composition of materials. It has been seen that the Ca/P ratio represents a characteristic biogenic signature of HA. Pure HA as previously reported, has a ratio of 1.62 [40,41,42]. In our study, the progression of the experiments displayed an increase in this ratio, starting at virtually null values, and achieving 1.23 for p-RWV, 1.14 for STAT and 0.77 for HARV, after 28 days. Although all bioprocesses produced non-stoichiometric HA, the increase is clear and more research should be performed until the proper ratio is reached.

Differentiation at a genetic level was assessed, examining the expression of Runx2/CBFA1, osteocalcin (BGLAP), alkaline phosphatase (ALPL), collagen type I (COL1A1) and sclerostin (SOST)—these genes are hallmarks of the entire MSC to osteocyte ontogenesis. Osteogenesis requires the precise and orchestrated activity of several genes and signals. Runx2 is a transcription factor and the central control. Its upregulation happens early, and it is believed to have a constant and low expression through the whole differentiation [43,44]. ALPL function is yet ill-defined. However, it is known that its action starts as early as 2 days after the onset of the process, with steadily increase through differentiation. Col1a1 is an important component in bone ECM, with a role in cell adhesion, proliferation and osteoblast phenotype—its upregulation is characteristic of osteogenic differentiation and can be considered an early marker. BGLAP is an osteoblast-specific gene, and its expression is significantly upregulated in matrix synthesis and mineralization [45]. Sclerostin is a known specific gene for mature osteocytes, and it is integral to osteocyte function as a signal to damp the action of osteoblast bone deposition and to control bone metabolism [34].

MSCs, pre-osteoblasts and osteoblasts express Runx2 together with secreting osteoid—when osteoblasts mature, they express alkaline phosphatase. Following maturity, osteoblasts become embedded in osteoid, and they express osteocalcin and start producing dendritic projections characteristic of osteocytes. The only known marker unique for osteocytes is sclerostin, which has been suggested to be partially responsible for lacunar formation and enlargement [34,46]. Perfusion culture showed a low and constant expression of RUNX2 along with an almost constant expression of osteocalcin. The expression of these genes, could suggest that most cells already went through the transition from immature to mature osteoblast [47]. The high increase in SOST in the last week could represent further transition from mature osteoblast-like to osteocyte-like cells. Nonetheless, to further corroborate this assertion, future studies incorporating additional markers such as DMP1 and MEPE would be required. During this period, Col1a1 and ALPL were active and positive. These genes may be indicative of a well-developed in vitro osteogenesis [48]. These results suggest that a homogeneous population of cells in the last stages of maturation was produced. HARV culture showed low enhancement in the expression of RUNX2 along with high increase in the expression of osteocalcin. This may indicate cells transitioning from immature cells to mature osteoblast. SOST expression in the late stage could suggest the presence of mature osteocyte-like cells. These results suggest heterogeneous populations of cells. 3D STAT showed a high increase in the expression of RUNX2 along with a relatively constant expression of osteocalcin. The expression of these two genes could represent the presence of a significant number of immature cells. The high SOST increase at the end of culture may represent that most cells that matured into osteoblast-like have further differentiated to osteocyte-like cells. These results suggest the production of a highly heterogeneous population [34].

P-RWV produced higher proliferation, calcification and a more homogeneous bone tissue. HARV produced a lower differentiation in comparison with the perfusion system and STAT showed good differentiation in the surface of the constructs, but poor in the inner part of the organoids. Additionally, cells left the construct to start growing on the flask surface.

Low proliferation of cells in HARV culture (compared with P-RWV) could have damped the differentiation—the morphogenetic analysis validates this idea and other authors have argued this could happen as a consequence of the collision of the beads with the wall of the reactor vessel, hurting cells in the surface of the scaffold and retarding differentiation [21]. However, this is not our case, as cells are grown in a free fall state. STAT produces a highly heterogeneous cell construct, with limited mineralization compared with the other bioprocesses, it is believed that mass transfer impediments produce poor nutrition of the cells and hinder the quality of the cell construct.

The enhanced proliferation, AP activity, OC secretion, and calcium deposition observed in the perfusion culture indicate a positive influence of the continuous feeding strategy, and potential mitigation of external mass transport limitations on the growth and differentiation of MSCs towards the osteoblastic phenotype.

## 4. Materials and Methods

### 4.1. Bioprocessing of hDPSCs

Traditional 2D static culture (STAT) has been able to find the principal cues for osteogenic differentiation, but has failed to yield the histo-architecture characteristic of bone. Hence, 3D scaffolds have been adopted as anchorage structures. In this platform, great improvements have been made. Proof of this is the vast variety of scaffolds that has been developed [49]. Scaffolds alone are considered insufficient to mimic the SC niche, as diffusion on its own is unable to fulfil the nutritional requirement of cells [50]. The dynamicity of bone transport characteristics has to be replicated. Accordingly, the design of adequate tailor-made bioprocesses able to achieve such benchmark is crucial [30,50,51,52]. Different bioprocess designs coupling bioreactors and perfusion have been able to produce advances in osteogenic neo-tissue formation. Consequently, these devices have potential to produce clinical hDPSC therapeutics with application in bone trauma and defect regeneration.

In this study, hDPSCs were encapsulated in alginate hydrogels and studied for long-term culture in vitro inside an in-house-designed dynamic perfused bioreactor system (P-RWV). The design provides a peristaltic pump to perfuse pre-oxygenated fresh media through a rotating vessel and a membrane vessel wall, for a second source of oxygenation. Here, cell-laden hydrogels are cultured, hence producing a suspension culture under low physiological fluid shear conditions. The dynamic environment inside the bioreactor has several advantages over static systems—enhanced transport characteristics, improving nutrient and oxygen availability inside the scaffolds and producing more homogeneous tissue [18,53,54]. Additionally, the fluid shear stress enhances osteogenic differentiation of hDPSCs. Shear stress affects cell growth and bone mechano-transduction, upregulating key osteogenic markers such as runt-related transcription factor 2 (CBFA1), alkaline phosphatase (ALP), osteocalcin (OCN) and osteopontin (OPN) [19]. A complex cascade of gene expression and cytokine/growth factor concentration leads the ontogenesis of a multipotent cell into an osteogenic cell, passing through osteoblast-like to a terminal-stage osteocyte-like cell. As a result, the different cells will produce a characteristic extracellular matrix (ECM) that will ultimately form the complex bone matrix.

We investigate the osteogenic differentiation capabilities of the P-RWV bioprocessing of hDPSCs and compare the results with a fed-batch high-aspect-ratio vessel bioreactor (HARV) and 3D static culture (3D STAT). A comprehensive characterization of the three systems was performed. The measurement of cell viability and the following up of bone ontogenesis was performed by monitoring structural and cellular changes by histology/immunocytochemistry and gene expression of markers characteristic of different stages of bone differentiation (undifferentiated, pre-osteoblast, osteoblast and osteocyte cells). The measurement of bone turnover was performed by examining the composition of the produced cell-laden scaffolds by different techniques. Our results provide a better understanding of the bioprocesses in question, along with the differentiation phenomenon itself.

### 4.2. hDPSC Extraction

hDPSCs were facilitated by Leeds Dental Institute, Leeds University (Leeds, UK) with full patient consent and ethical approval (LREC 07/H1306/93). Patients (16–25 years old) were asked to voluntarily sign informed consent. Isolation of hDPSCs was carried out according to Tirinio et al. [55]. In short, extracted teeth were split, pulp tissue was removed, minced and digested in a mixture of 3 mg/mL type I collagenase and 4 mg/mL dispase (Sigma-Aldrich, Poole, UK) at 37 °C for 30–60 min. Once digested, the mixture was filtered through a 70 μm cell strainer and centrifuged (1500 rpm, 10 min). Detached cells were plated in a 35 mm dish containing minimum essential medium Eagle–alpha modification (αMEM, Gibco, NY, USA) with 10% Fetal Bovine Serum (FBS, Gibco, NY, USA), 2 mM L-glutamine (L-GLu, Gibco, NY, USA), 0.1 mM L-Ascorbic Acid 2-Phosphate (L-ASAP, Sigma-Aldrich, Poole, UK) and 100 U/mL Penicillin/Streptomycin antibiotics (Pen/Strep, Gibco, NY, USA), and incubated at 37 °C in a 5% CO_2_ incubator until 80% confluence.

### 4.3. Human Dental Pulp Stem Cell Culture

Cells were cultured on T-75 tissue culture flasks as previously reported [56] and 3D cultures were established with passage 4 cells. For encapsulation, cells were detached from T-flasks with TrypLE express (Thermo Fisher scientific), counted and resuspended at a density of 2.5 × 10^6^ cells/mL in a solution of 1.1% (*w*/*v*) alginic acid (Sigma) and 0.1% (*v*/*v*) gelatin (Sigma) in PBS (pH 7.4) as described in our previous studies [18,20]. The cell suspension was driven through a peristaltic pump (Model P-1, Amesham Biosciences, U.K.) and dropped from at least 30 mm height into gently stirred crosslinking solution (100 mM calcium chloride (CaCl2) and 10 mM N-(s-hydroxyethyl) piperazine-N-(2-ethane sulfonic acid) at pH 7.4). The flow rate of the pump was adjusted to give single droplets using a 25-gauge needle (Becton Dickinson, Oxfordshire, UK). Upon contact with the solution, gelling of the alginate droplets started and after 5 min in the solution, bead-shaped gels with an approximate diameter of 1.3 mm were obtained. Organoids were acclimatized to the 3D environment and expanded during 7 days of culture in the maintenance medium consisting of the minimum essential medium Eagle–alpha modification (αMEM, Gibco, NY, USA) with 10% Fetal Bovine Serum (FBS, Gibco, NY, USA), 2 mM L-glutamine (L-GLu, Gibco, NY, USA), 0.1 mM L-Ascorbic Acid 2-Phosphate (L-ASAP, Sigma-Aldrich, Poole, UK) and 100 U/mL Penicillin/Streptomycin antibiotics (Pen/Strep, Gibco, NY, USA). Osteogenic differentiation was achieved by culturing for the following 21 days in the osteogenic medium consisting of the maintenance medium plus 5 mM β-glycerophosphate (β-GP [20], Sigma-Aldrich, Poole, UK), 0.1 mM dexamethasone (Dex, Sigma-Aldrich, Poole, UK), 0.5 mM L-ASAP (Sigma-Aldrich, Poole, UK), and 1.8 mM KH_2_PO_4_ (Sigma-Aldrich, Poole, UK). Three different culture systems were established and compared: (a) static performed in 175 Corning flasks (3D STAT); (b) batch cultures, in commercially available 55 mL high-aspect-ratio vessel (HARV) synthecon bioreactors (Cellon, Bascharage, Luxembourg); and (c) perfusion cultures, in an in-house-designed perfusion bioreactor (P-RWV), which consisted of an external oxygenator, composed of a spiral roll of gas-permeable silicon tubing with a 3 mm internal diameter, a wall thickness of 0.5 mm, and approximately 3.4 m in length, and a 60 mL vessel fabricated using a dual-sided silicone-polytetrafluoroethylene (PTFE) gas-permeable membrane (Specialty Silicone Products inc., Ballston Spa, NY, USA). Pictures of the three culture systems can be seen in the Supplementary Materials (Figure S1). The three culture systems contained approximately 250 beads per vessel and each experiment was performed in triplicate. Bioreactors were placed in 20% oxygen and 5% CO_2_ conditions. Batch cultures (3D STAT and HARV) were fed once a day for the 28-day culture period, whereas the medium was continuously supplied at 2.29 mL/hr in perfusion culture conditions (P-RWV, one vessel per day). Macroscopic images of organoids at day 0 and 28 of culture can be seen in the Supplementary Materials provided.

### 4.4. Live/Dead Assay

Live/dead staining for the hydrogels was conducted in situ, as described previously [57]. Briefly, beads were incubated at 37 °C for 30 min in the dark using the live/dead cytotoxicity kit consisting of 4 mM EthD-1 and 2 mM calcein AM solution (Invitrogen, Paisley, UK). Following staining, hydrogels were washed thoroughly with PBS and imaged within 30 min. Images were captured using an Olympus BX51 microscope (Olympus, Tokyo, Japan) and F-view unit (Olympus Soft Imaging Solurtions GmbH, Münster, Germany).

### 4.5. Cell Counting Kit 8 Viability Assay (MTS)

Total cell numbers were obtained using Cell counting kit 8 (CCK-8; Sigma-Aldrich, Poole, UK) as per the manufacturer’s instructions and analyzed measuring absorbance at 450 nm with a GLoMax 96 microplate luminometer (Promega, WI, USA). Briefly, beads were harvested and deposited in 96 well-plates with 100 uL of media. Samples were pre-incubated at 37 °C and 5% CO_2_. A volume of 10 uL of CCK-8 reagent was added to each well and incubated for 4 h. After, the supernatant absorbance was analyzed.

### 4.6. qPCR

Total RNA was extracted from collected cells using the RNeasy kit (Qiagen, West Sussex, UK) as per the manufacturer’s instructions and quantified using a BioPhotometer plus (Eppendorf UK, Cambridge, UK). Relative gene expression was analyzed by conducting real-time quantitative polymerase chain reaction (qPCR) on genes of interest. The SensiMIX™ SYBR No-ROX One-Step kit (Bioline, London, UK) was used, which combines cDNA synthesis with PCR amplification within a single assay per the manufacturer’s instructions. Each PCR reaction consisted of 0.2 µM of primer (sense/anti-sense), 12 units of RNase inhibitor, 80 ng of total RNA and 1× SensiMix SYBR solution containing SYBR^®^ green I dye dNTPs, reverse transcriptase, hot-start DNA polymerase, SensiTaq and 3 mM MgCl. Relative gene expression analysis was conducted using the 2^−ΔΔCT^ method [58] to calculate the relative fold differences between the normalizing value (T = 0, beginning of experiment) and glyceraldehyde 3-phosphate dehydrogenase (GAPDH) as the reference gene. Primer details are summarized in Table 1.

### 4.7. Immunocytochemical Staining

Hydrogels were fixed for 90 min in 4% (*w*/*v*) paraformaldehyde (BDH Laboratory supplies, Dubai, UEA) at room temperature (RT), washed with rinse buffer, 20 mM Tris-HCl buffer (Sigma-Aldrich, Poole, UK), 0.15% NaCl and 0.05% Tween-20 (Sigma-Aldrich, Poole, UK) and placed into tubes. Samples were then permeabilized-blocked by incubating for 45 min in permeabilization-blocking solution. A good blocking solution consists of the serum of the same animal species rather than the host of the secondary antibody—in this study, rabbit and goat serum were used. The permeabilization-blocking solution was removed and samples were washed 2 times in rinse buffer. Samples were incubated overnight in a humid chamber with the primary antibodies diluted in 0.1% triton and 1% BSA in PBS—for the control sample, only diluent was added. The solution was removed and washed twice with rinse buffer 1X. Samples were then incubated for 1 h in the dark at room RT, with the corresponding secondary antibodies diluted in PBS (including the control). From this step onwards, everything was performed in darkness. The secondary antibody solution was removed and samples were washed in rinse buffer 1X. The buffer was removed and samples were observed under the microscope.

### 4.8. Histochemistry Staining

Hydrogels were fixed with 10% (*v*/*v*) paraformaldehyde (Sigma-Aldrich, Poole, UK) for 30 min at room temperature and then placed in PBS (Sigma-Aldrich, Poole, UK) for 15 min. Dehydration was achieved by placing in a sequential series of increasing ethanol concentrations. Ethanol was completely replaced with increasing xylene concentration solutions followed by a 100% xylene (Sigma-Aldrich, Poole, UK) step prior to incubation with paraffin-saturated xylene at RT overnight. Hydrogels in paraffin-saturated xylene were then placed in an oven (60 °C) for 20 min to remove xylene. Hydrogels were then serially sectioned (4 μm) and left at RT overnight to adhere to Vectabonded™ (Vector Laboratories, Newark, CA, USA) glass slides. Paraffin was completely removed by immersion in xylene, decreasing ethanol concentrations and then by washing with tap water.

For haematoxylin & eosin (H&E) staining, slides were dipped in haematoxylin (Harris’ solution; BDH Laboratory Supplies, Dubai, UAE) for 30 s following fixation. The slides were washed in running tap water for 2–3 min and examined for sufficient staining before being dipped in 1% eosin (BDH Laboratory Supplies, Dubai, UAE) for 2 min and washed briefly in tap water. For Alizarin red S (ARS) staining, slides were immersed in a 2% (*w*/*v*) ARS (Sigma-Aldrich, Poole, UK) solution (pH 4.2) for 5 min, washed in tap water, and counterstained with Mayer’s haematoxylin. Images were captured using an Olympus DP50 digital camera (Olympus, Tokyo, Japan) and analyzed using analySIS^®^ imaging software, version 3.1 (Soft Image System GmbH, Muenster, Germany).

For Von Kossa staining (VK, Sigma-Aldrich, Poole, UK), slides were immersed for 20 min in 1% silver nitrate solution under ultraviolet light and then rinsed in several changes in distilled water. Following this, slides were immersed in 5% sodium thiosulfate for 5 min. Nuclear fast red was used as a counterstain.

### 4.9. Alkaline Phosphatase (ALPase) Activity

Cellular ALPase was assessed colorimetrically by using alkaline phosphatase assay kit (Sigma-Aldrich, Poole, UK) as per the manufacturer’s instructions. Briefly, beads were collected from each group, 200 µL of ALPase buffer and 200 µL of rNPP were added to each sample and incubated at 37 °C for 30 min in the dark. To stop the reaction, 400 µL of 0.5 N sodium hydroxide was added to the samples. Finally, 100 µL of the solution was analyzed at 410 nm wavelength with ELISA reader (MRX II plate reader; Dynex technologies, West Sussex, UK).

### 4.10. Scanning Electron Microscopy with X-Ray Microanalysis

Organoids were fixed with 3% glutaraldehyde solution (Sigma-Aldrich, Poole, UK) in Sorenson’s buffer and washed with Sorenson’s buffer for 5 min. Post-fixation was conducted with 4% osmium tetroxide (Sigma-Aldrich, Poole, UK) in a cold bath for 15 min. Beads were rinsed carefully in Sorenson’s buffer to avoid structural damage of samples. Samples were dried in a series of increasing concentrations of ethanol and mounted on stubs. Mounted samples were coated with gold in a sputter coater and then viewed. Digital image capture was performed in Jeol JSM5610LV SEM (Jeol UK Ltd, Hertfordshire, UK) and x-ray microanalysis was performed in Jeol JSM6400 SEM fitted with Oxford Instruments INCA energy dispersive analytical system (EDS) for elemental x-ray.

### 4.11. Attenuated Total Reflectance–Fourier Transform Infrared Spectroscopy

Alginate organoids were characterized by microattenuated total reflectance–Fourier transform infrared (ATR-FTIR) spectroscopy. FTIR images were collected with a setup described previously [59] using a diamond ATR accessory (Specac Ltd, Kent, UK) and a 64 × 64 focal plane array detector (FPA). The imaged area was 500 × 700 μm^2^. All images were taken with a spectral resolution of 8 cm^−1^ in the range 4000–900 cm^−1^ using 64 co-adding scans. The chemical images showing the distribution of hydroxyapatite (HA) developed in the organoids were extracted using factor analysis to the spectral region of 1110–990 cm^−1^.

### 4.12. Media Analysis

The concentration profile of nutrients (glucose, glutamine, lactate, ammonia, glutamate) and pH were measured using Bioprofile 400 Analyzer (Nova Biomedical, Flintshire, UK) taking 1.0 mL samples of culture media supernatant to be harvested at several time points during the culture. The fresh medium was used as the control.

### 4.13. Statistical Analysis

Samples for quantitative analyses were measured in three replicates. Comparable values from each group were subjected to statistical analysis with Student’s *t*-test or analysis of variance (ANOVA) with a significance of *p* < 0.05 (*). Table 1 shows primers for osteogenic differentiation characterization.

## 5. Conclusions

This study demonstrated that bioprocessing providing perfusion flow of the medium in a suspended environment is successful in mitigating nutrient and oxygen transport limitations, external to three-dimensional hDPSCs/alginate constructs and is more efficient than HARV and 3D STAT. This positively influences the proliferation, differentiation, mineralization and expression of osteoblastic markers of hDPSCs, ultimately enabling the formation of 3D bone-like constructs for clinical purposes. Progressive differentiation was confirmed for the three different bioprocesses by their gene expression patterns, the increase and further decrease in ALPase activity, the gradual decrease in the C/P ratio, the increase in the Ca/P ratio and the ARS-positive area. The perfusion system achieved better results in most of the analysis, producing a homogeneous cell construct with potential use in hDPSC-based application in regenerative medicine. In contrast, STAT produced a highly heterogeneous cell construct and due to mass transfer limitations, resulted in an unfavorable environment for cells to grow and differentiate. HARV demonstrated slower proliferation than the perfusion culture, which may have delayed or dampened the normal differentiation pattern of osteoblast-like cells.

The perfusion culture system described here would provide a scalable, efficient, and easy culture system for applications of hDPSCs in BTE in the context of macroscopic bone formation.

## Figures and Tables

**Table 1:** Primers for osteogenic differentiation characterization.

Gene	Sequence		Size	T_m_ (°C)
GAPDH	Forward Primer	GGAGCGAGATCCCTCCAAAAT	197	62
	Reverse Primer	GGCTGTTGTCATACTTCTCATGG		
Runx2	Forward Primer	TGGTTACTGTCATGGCGGGTA	101	62
	Reverse Primer	TCTCAGATCGTTGAACCTTGCTA		
Col1a1	Forward Primer	GAGGGCCAAGACGAAGACATC	140	62
	Reverse Primer	CAGATCACGTCATCGCACAAC		
ALPL	Forward Primer	ACCACCACGAGAGTGAACCA	79	62
	Reverse Primer	CGTTGTCTGAGTACCAGTCCC		
SOST	Forward Primer	ACACAGCCTTCCGTGTAGTG	123	61
	Reverse Primer	GGTTCATGGTCTTGTTGTTCTCC		
BGLAP	Forward Primer	CACTCCTCGCCCTATTGGC	112	62
	Reverse Primer	CCCTCCTGCTTGGACACAAAG		

## Data Availability

The data that support the findings of this study are not openly available due to reasons of sensitivity and are available from the corresponding author upon reasonable request.

## Supplementary Materials

The following supporting information can be downloaded at: https://www.mdpi.com/article/10.3390/ijms26094348/s1.
