# Supplementary material for: Engineering an Integrated Bioprocess to Produce Human Dental Pulp Stem Cell-Alginate-Based Bone Organoids
Source: Int J Mol Sci. 2025 May 3;26(9):4348. doi: 10.3390/ijms26094348 (PMC12073084; doi:10.3390/ijms26094348)
Supplement: Supplementary file 1 [file ijms-26-04348-s001.zip › ijms-3572165-supplementary.pdf]

Supplementary material

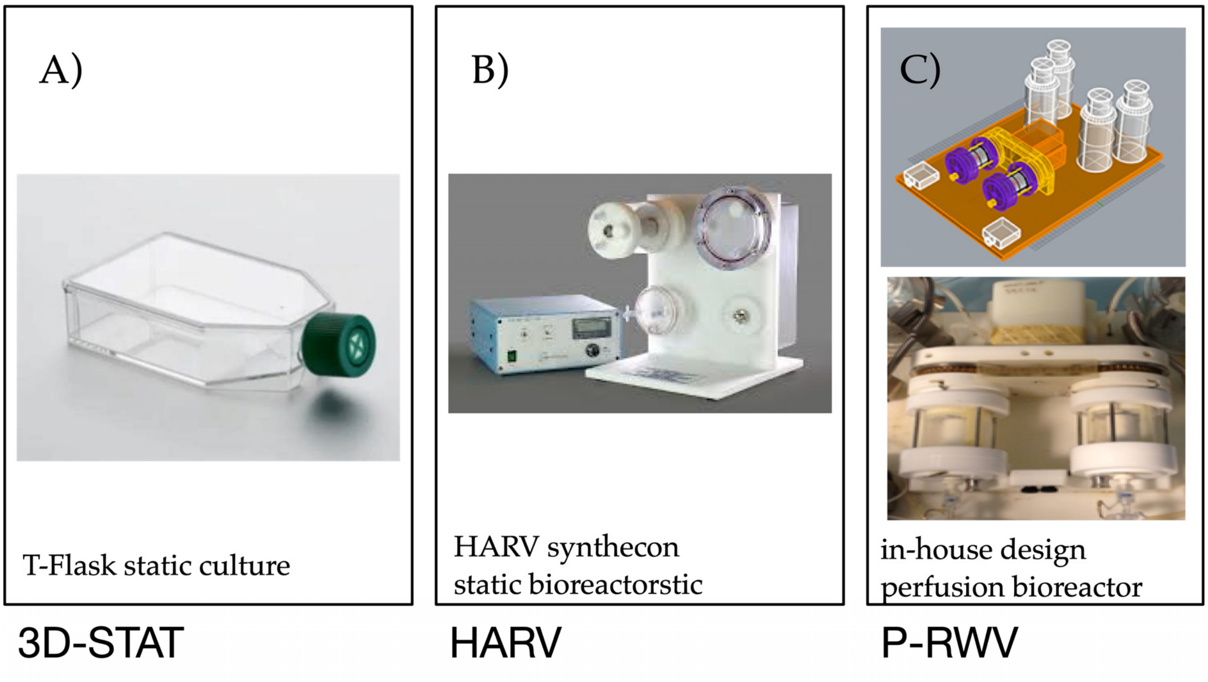

Figure S1. a) T-Flask static culture, b) HARV Synthecon bioreactor, and a) Dual chambered in-house-designed perfusion rotating wall vessel bioreactor (P-RWV)

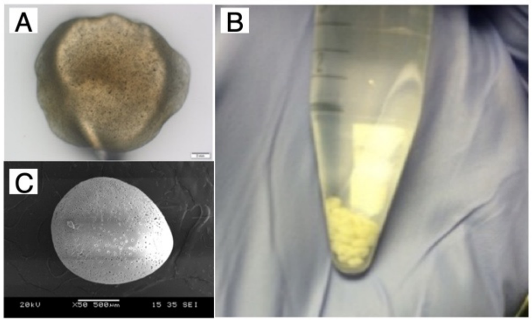

Figure S2. Macroscopic images of the organoids, A displays hidrogels at day 0, B at day 28, and C is a SEM image at day 28. Organoid size, app 2.3 mm.

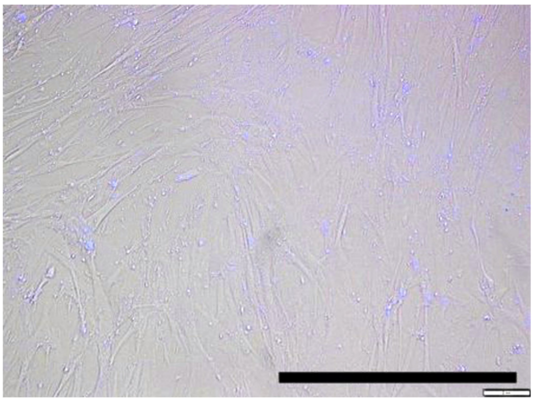

Figure S3. hDPSCs adhered to culture flask by day 14 of culture in 3D STAT, these cells left the scaffold as consequence of mass transport limitations. Scale bar=200 µm.

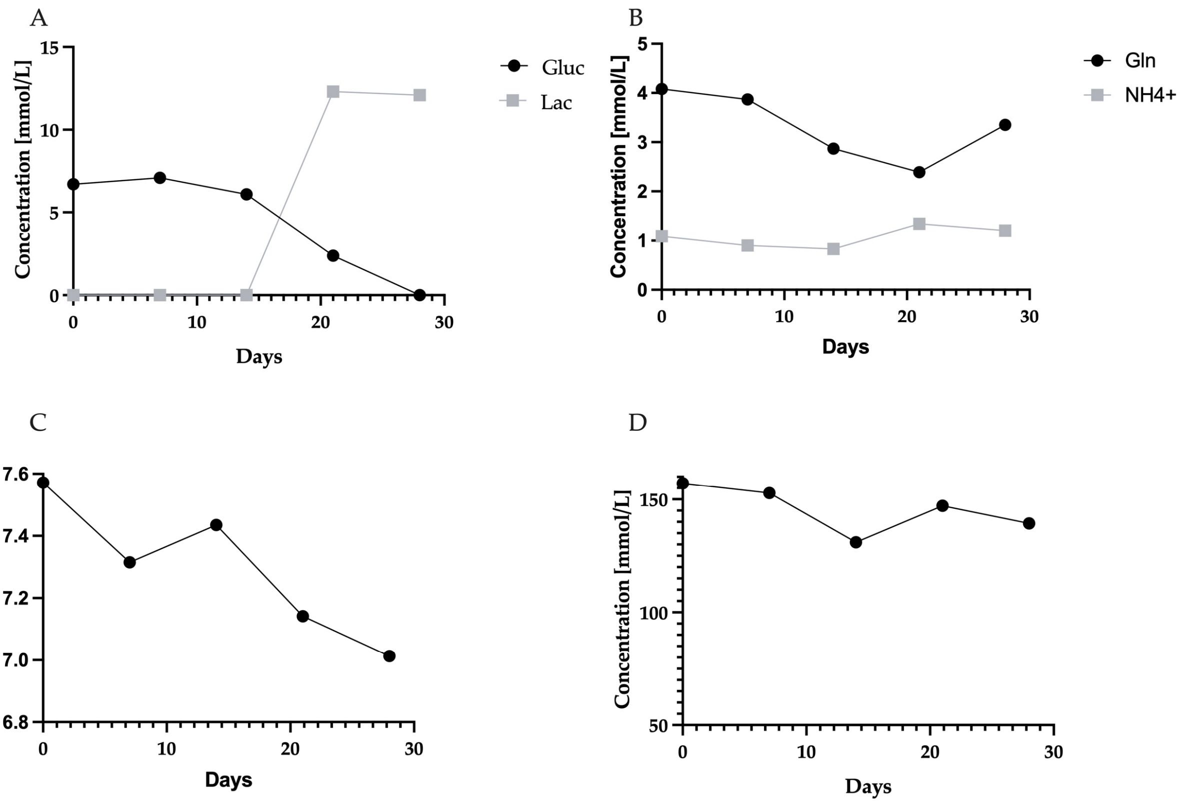

Figure S4. Change with culture time of key substrates and metabolites in culture medium for 3D STAT. A) Consumption of glucose and production of lactate. B) Consumption of glutamine and production of ammonia. C) pH. And D) Oxygen.

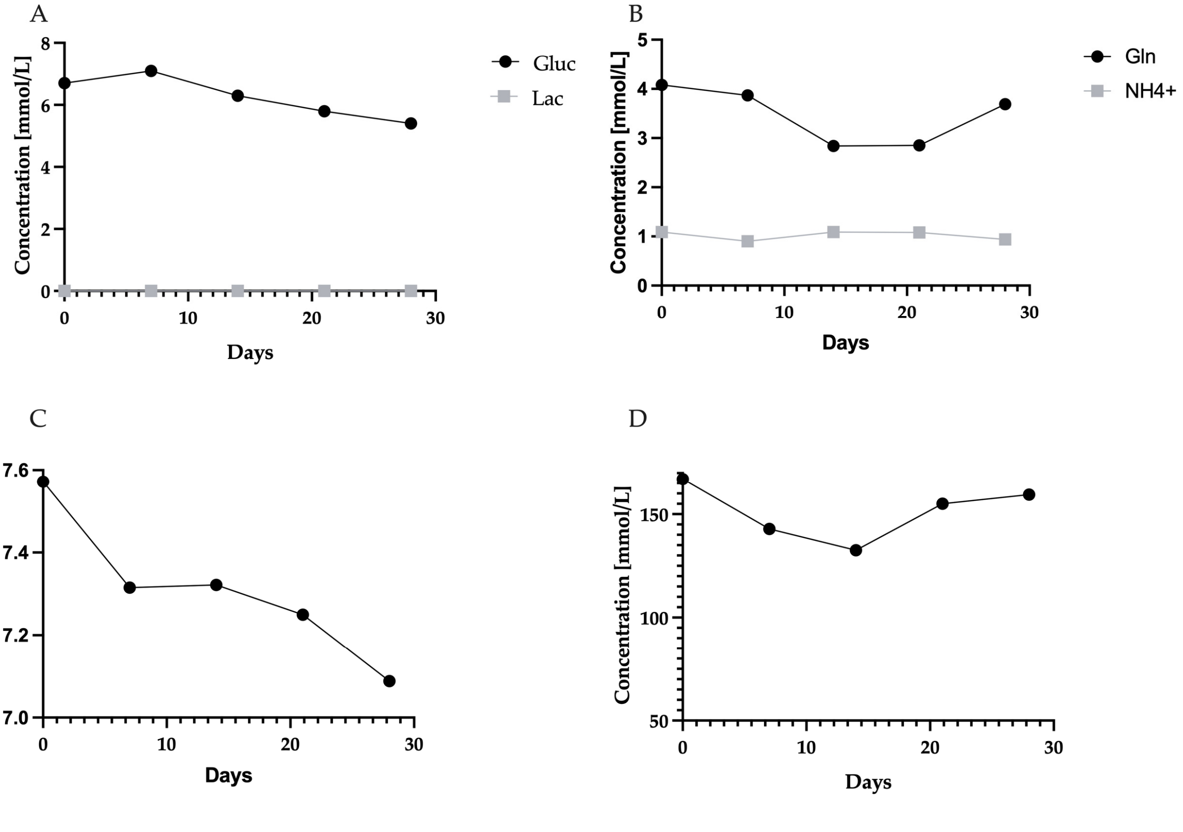

Figure S5. Change with culture time of key substrates and metabolites in culture medium for HARV. A) Consumption of glucose and production of lactate. B) Consumption of glutamine and production of ammonia. C) pH. And D) Oxygen.

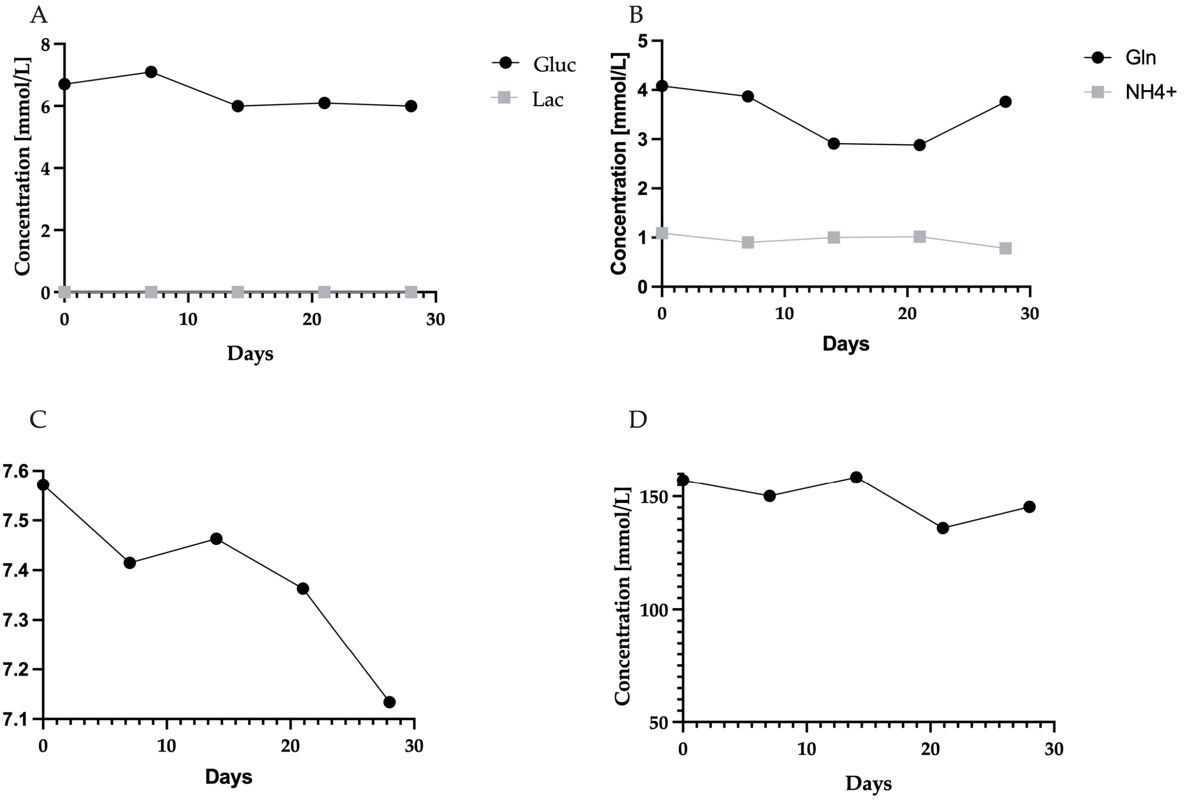

Figure S6. Change with culture time of key substrates and metabolites in culture medium for P-RWV. A) Consumption of glucose and production of lactate. B) Consumption of glutamine and production of ammonia. C) pH. And D) Oxygen.
